# Cannabis use is associated with a small increase in the risk of postoperative nausea and vomiting: a retrospective machine-learning causal analysis

**DOI:** 10.1186/s12871-020-01036-4

**Published:** 2020-05-18

**Authors:** Wendy Suhre, Vikas O’Reilly-Shah, Wil Van Cleve

**Affiliations:** 1 Department of Anesthesiology and Pain Medicine, University of Washington, Box 356540, 1959 NE Pacific St, Seattle, WA 98195 USA; 2 Perioperative & Pain Initiatives in Quality, Safety, and Outcome, Department of Anesthesiology and Pain Medicine, University of Washington, Box 356540, 1959 NE Pacific St, Seattle, WA 98195 USA; 3 Seattle Children’s Hospital, 4800 Sand Point Way, Seattle, WA 98105 USA

**Keywords:** Cannabis, Postoperative nausea and vomiting, Cross-sectional studies, Machine learning

## Abstract

**Background:**

Cannabis legalization may contribute to an increased frequency of chronic use among patients presenting for surgery. At present, it is unknown whether chronic cannabis use modifies the risk of postoperative nausea and vomiting (PONV).

**Methods:**

This study was a retrospective cohort study conducted at 2 academic medical centers. Twenty-seven thousand three hundred eighty-eight adult ASA 1–3 patients having general anesthesia for non-obstetric, non-cardiac procedures and receiving postoperative care in the Post Anesthesia Care Unit (PACU) were analyzed in the main dataset, and 16,245 patients in the external validation dataset. The main predictor was patient reported use of cannabis in any form collected during pre-anesthesia evaluation and recorded in the chart. The primary outcome was documented PONV of any severity prior to PACU discharge, including administration of rescue medications in PACU. Relevant clinical covariates (risk factors for PONV, surgical characteristics, administered prophylactic antiemetic drugs) were also recorded.

**Results:**

10.0% of patients in the analytic dataset endorsed chronic cannabis use. Using Bayesian Additive Regression Trees (BART), we estimated that the relative risk for PONV associated with daily cannabis use was 1.19 (95 CI% 1.00–1.45). The absolute marginal increase in risk of PONV associated with daily cannabis use was 3.3% (95% CI 0.4–6.4%). We observed a lesser association between current, non-daily use of cannabis (RR 1.07, 95% CI 0.94–1.21). An internal validation analysis conducted using propensity score adjustment and Bayesian logistic modeling indicated a similar size and magnitude of the association between cannabis use and PONV (OR 1.15, 90% CI 0.98–1.33). As an external validation, we used data from another hospital in our care system to create an independent model that demonstrated essentially identical associations between cannabis use and PONV.

**Conclusions:**

Cannabis use is associated with an increased relative risk and a small increase in the marginal probability of PONV.

## Background

Medicinal use of cannabis was first described in 1840 by W.B. O’Shaughnessy, a medical doctor and chemist in Calcutta, who described its use for the treatment of acute and chronic rheumatism, rabies, tetanus, cholera, and infantile convulsions [1]. Cannabis is currently classified as a Schedule 1 drug in the United States, a classification for drugs considered by the Drug Enforcement Agency to have no accepted medical use and an unacceptable risk of abuse [2]. Beginning in 1996, a gradual process of cannabis legalization has taken place in the US, with 33 states as well as the District of Columbia permitting medical use and 14 US states and territories presently allowing recreational use of cannabis [3]. In Washington State, where this research was conducted, recreational use of cannabis by adults 21 years of age and older was legalized in 2012.

In the nineteenth century, Dr. O’Shaughnessy described the use of hemp seeds to treat many diseases, and specifically noted that they “allayed vomiting” in cholera patients. Today, the cannabinoids present in cannabis are used in a medical context to treat various medical conditions, among them chemotherapy induced nausea and vomiting (CINV). Multiple studies using synthetic cannabinoids have shown cannabis to be as effective as other antiemetics for this purpose [4–6].

As cannabinoid compounds have been shown to be effective treatments for CINV, it seems reasonable to conjecture that cannabis use could exert a prophylactic or therapeutic effect for patients at risk for or suffering from postoperative nausea and vomiting (PONV). While several studies have examined the role of therapeutically administered cannabinoids in the prevention and treatment of PONV, almost nothing is known about the impact of chronic use of cannabis on the risk for developing PONV [7–10]. The present investigation examines whether an association exists between patient-described use and/or frequency of cannabis and the occurrence of PONV following general anesthesia.

## Methods

This study was a retrospective cohort analysis of general anesthesia cases lasting 30 min or longer conducted at the University of Washington Medical Center (UWMC) from July 1, 2016 until September 30, 2018. Data from Harborview Medical Center (HMC) from the same time period were used for model validation. Inclusion criteria were general anesthesia cases for patients aged 18 years and older with a documented pre-anesthetic evaluation who also received post-operative care in the Post Anesthesia Care Unit. Data regarding anesthetic management were obtained from the hospital Anesthesia Information Management System (Merge AIMS, Hartland, WI). Obstetric and cardiac cases were excluded, as were cases with an American Society of Anesthesiologists Physical Classification 4 or greater. Data regarding risk factors for PONV and pattern of ongoing cannabis use were gathered from the pre-anesthetic evaluation documented for the case. Data regarding the occurrence of PONV were abstracted automatically from nursing documentation in the post-anesthesia care unit. Severity of PONV was not considered in this analysis. The dataset was obtained from a central repository of perioperative and anesthetic data maintained by the UW Perioperative and Pain initiatives in Quality and Safety Outcome Center, which performed data extraction, validation, and de- identification prior to providing it to our research team. Because of patient de-identification, this study was exempted from review by the University of Washington Institutional Review Board as non-human subjects research. This manuscript was prepared in accordance with STROBE guidelines for improved reporting of observational studies [11].

### Primary predictor

Plain text from the preoperative evaluation note regarding the use of non-prescribed substances/drugs was extracted and manually reviewed by one of the investigators (WS). cannabis use as described by the patient was classified by the investigator as “daily” (used on a daily basis), “current” (used at present, but less often than daily) or “none” (i.e. past use was not considered).

### Primary outcome

A composite variable constituted by PONV of any severity as recorded by the recovery room nurse, or the administration of an antiemetic drug in the PACU (ondansetron, promethazine, perphenazine, or metoclopramide), was used to indicate the presence of PONV in our analysis.

### Covariates

Following the strategy employed by a recent published study examining associations between perioperative medication use and PONV, we picked a set of a priori covariates we expected to be associated with PONV [12]. These included (a) age less than 50 years, (b) ASA classification, (c) exposure to nitrous oxide (defined as exposure to nitrous oxide for greater than 5% of surgical time), (d) exposure to a potent volatile anesthetic agent (defined as age adjusted MAC > 0.5 for greater than 15% of surgical time), (e) surgical duration in minutes (log transformed), (f) female sex, (g) history of PONV or motion sickness, (h) absence of patient reported tobacco use, (i) receipt of an opioid drug in the PACU, and (j) the total number of prophylactic anti-emetic drugs given pre- or intraoperatively (drugs considered included dexamethasone, gabapentin, haloperidol, meclizine, metoclopradmide, ondansetron, prochlorperazine, promethazine, and transdermal scopolamine). Notably, many of these covariates were unlikely to be associated with both cannabis use and PONV, classifying them as potential effect modifiers rather than confounders. In some of our analyses, we combined the PONV risks commonly summed to create the “simplified Apfel score” (i.e. female sex, history of PONV or motion sickness, absence of patient reported tobacco use, and receipt of an opioid drug in the PACU) and stratified our analysis by the number of PONV risks [13, 14].

### Statistical analysis

Our primary analysis estimated the causal effect of cannabis use on PONV. Realizing that cannabis use was not randomly distributed throughout our sample, we employed a statistical method known as Bayesian Additive Regression Trees (BART). BART combines flexible nonparametric regression tree methods with a “Bayesian backfitting” algorithm that minimizes the amount of overfitting that can occur in similar machine learning algorithms [15]. BART has been demonstrated to generate valid causal effect estimates without the well-described weaknesses of propensity score estimation or matching, which include the potential for improper specification of the propensity model, problems handling large numbers of covariates, and proper modeling of non-linear relationships and variable interactions [16].

Analyses were performed using R version 3.6.2 (R Core Team, Vienna, Austria) within the RStudio platform 1.2.1335 (R Studio Team, Boston, MA). Probit BART models for the primary analysis were created using the BART package v2.7 [17]. We calculated 2 formulations of the causal effect estimate: the relative risk of PONV and an absolute increase in the probability of PONV associated with no use of cannabis (referent group), current use, and daily use. Counterfactual sample estimates were generated by artificially assigning all members of the sample to each condition and comparing the probability of the outcome of interest under each condition, allowing us to calculate the sample average treatment effect (sATE). Because BART provides a true Bayesian posterior estimate, we generated 95% credible intervals by carrying out the aforementioned analyses for each of 1000 Markov Chain Monte Carlo (MCMC) estimates, and then extracting the appropriate quantile from the resulting population of parameter estimates. As an internal validation of our initial result, we performed a propensity score analysis: we first created a Bayesian logistic regression model to model the probability of using any cannabis using the brms package v 2.11.1, followed by a second Bayesian logistic regression model that included our estimated probability of cannabis use as a covariate (e.g. propensity score adjustment) alongside parameters otherwise identical to those in our BART model [18]. We then examined both the parameter estimates and sample average treatment effects of this model [18, 19]. Finally, as an external validation of our findings, we created a second BART model with parameters identical to those used in our initial model using data collected at HMC, and again assessed the sATE for cannabis use on the risk of PONV. All statistical analyses were conducted by the primary research team.

### Statistical significance

Bayesian posterior estimates differ fundamentally from frequentist parameter summaries, and therefore no a priori statement about binary *p*-value thresholds representing statistical significance can be offered. We report 95% credible intervals for our parameter estimates, which represent the numeric interval in which 95% of the posterior probability density lies. Further, when estimating relative risk, we calculated the posterior probability that the relative risk exceeded 1.

## Results

After applying inclusion and exclusion criteria, 27,388 unique anesthetics at UWMC were available for analysis (Table 1). When stratified by self-described cannabis use, a higher proportion of daily users were ASA 3 (58%) than non-users (42.7%). Considering risk factors for PONV: daily cannabis users were more often male and more likely to smoke tobacco, but also had higher rates of prior PONV/motion sickness and higher rates of opioid use in the PACU when compared to non-users. The unadjusted incidence of PONV was higher in daily users (21.9%) and current users (18.8%) when compared to non-users (17.3%).

**Table 1:** Demographic and clinical data for general anesthetics at UWMC. Continuous variables are summarized by mean (sd). Categorical variables are summarised by n and %. Ordinal variables are summarized by median and interquartile range

*Variable*	Cannabis Use		
	None	Current	Daily
n (%)	24,662 (90.0)	1976 (7.2)	750 (2.7)
**Preoperative Data**			
Age in years [mean (sd)]	53 (16)	48 (15)	50 (14)
ASA [n (%)]			
1	2577 (10.4)	176 (8.9)	19 (2.5)
2	11,533 (46.8)	925 (46.8)	292 (38.9)
3	10,532 (42.7)	875 (44.3)	439 (58.5)
Outpatient [n (%)]	16,321 (65.6)	1212 (61.3)	438 (58.4)
Male Sex [n (%)]	10,833 (44.3)	1055 (53.4)	390 (52.0)
Non-smoker [n (%)]	22,571 (92.3)	1526 (77.2)	525 (70.0)
Prior PONV/Motion Sickness [n (%)]	4604 (18.3)	368 (18.6)	191 (25.5)
**Intraoperative Data**			
Procedure Duration (min) [mean (sd)]	120 (94)	126 (105)	133 (110)
Exposed to Nitrous Oxide [n (%)]	3152 (12.8)	240 (12.1)	111 (14.8)
Surgery Higher Risk for Nausea [n (%)]	4425 (17.5)	341 (17.3)	146 (19.5)
Total Number of Prophylactic Agents (median, [IQR])	2 [2,3]	2 [2,3]	2 [1,2]
**Postoperative Data**			
PACU Opioids [n (%)]	12,969 (52.6)	1184 (59.9)	476 (63.5)
Apfel Score (median, [IQR])	2 [2,3]	2 [1,3]	2 [1,3]
**Outcome**			
PONV Observed [n (%)]	4255 (17.3)	372 (18.8)	164 (21.9)

**Fig. 1:**
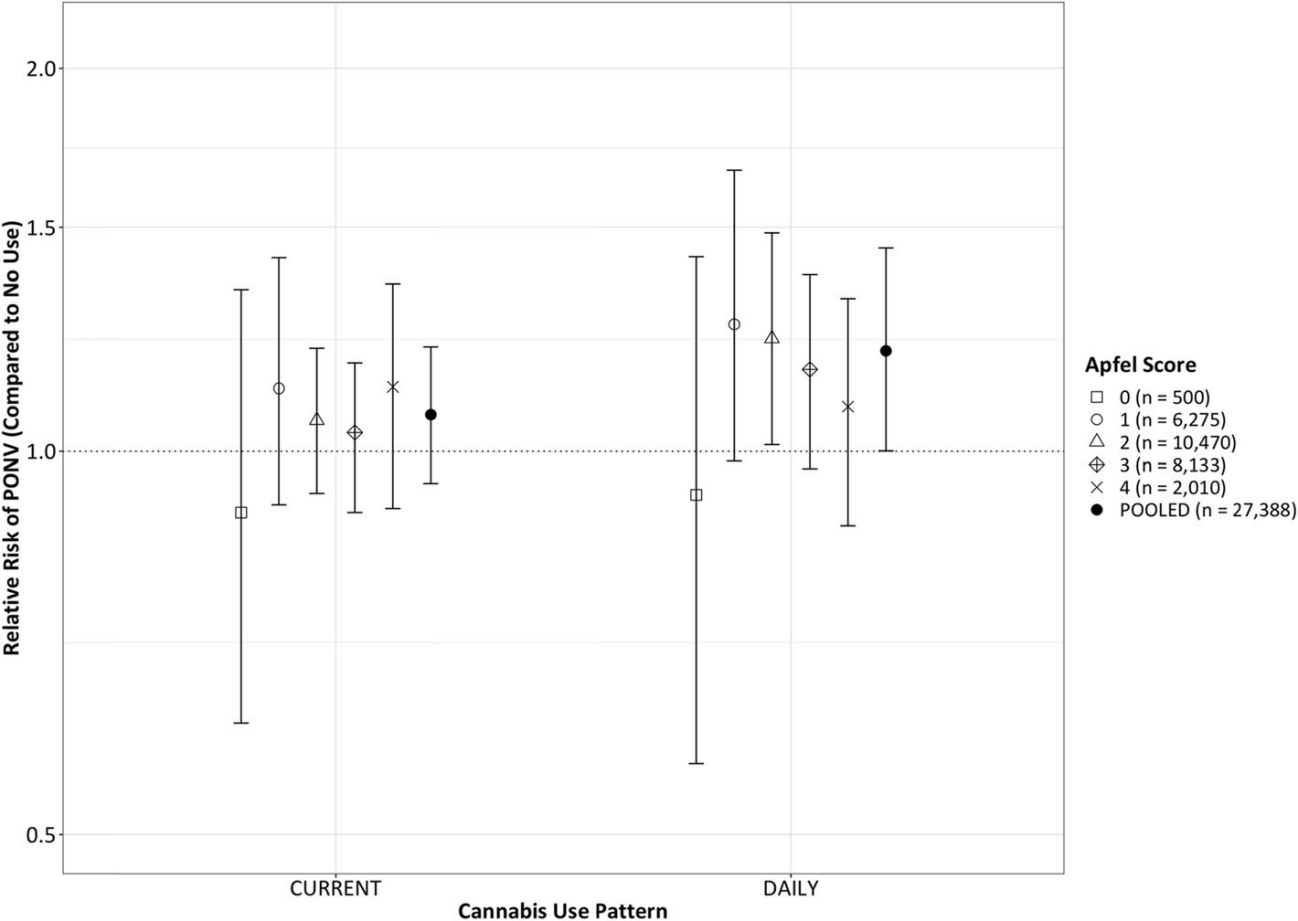
Sample average treatment effect (SATE) measured as relative risk of any postoperative nausea/vomiting modeling entire sample as non-users, current (non-daily), or daily cannabis users. Estimates stratified by Apfel score. 95% Bayesian posterior credible interval for SATE generated from 1000 MCMC estimates. Pooled estimate across all Apfel scores showed at right of each grouping

**Fig. 2:**
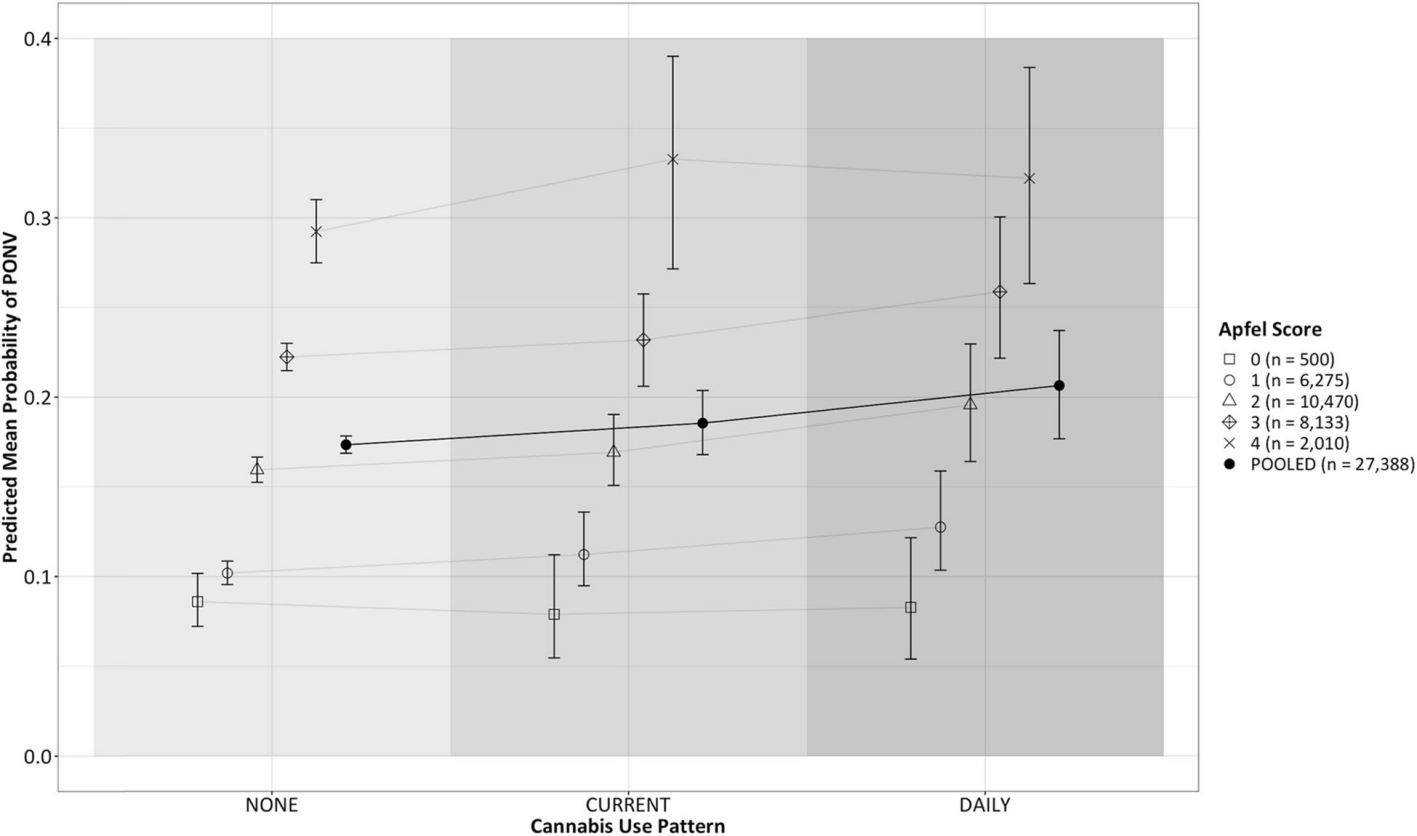
Sample mean predicted probability of PONV of postoperative nausea/vomiting stratified by Apfel score and conditioned on pattern of cannabis use. 95% Bayesian posterior credible interval for mean probability generated from 1000 MCMC estimates. Pooled mean probability estimate across all Apfel scores showed at right of each grouping

A probit BART model was created to model the probability of any PONV or rescue administration in the recovery room. Graphical depiction of the results of this model are provided in Fig. 1 and Fig. 2. The pooled relative risk of PONV was higher in daily users when compared to non-users, with a relative risk of 1.20 (95% CI 1.00–1.45, posterior probability RR > 1 = 97.6%), and slightly higher in current users compared to non users, with a relative risk of 1.07 (95% CI 0.94–1.21, posterior probability RR > 1 = 84.7%). As can be observed in Fig. 1, the increased probability of PONV associated with daily cannabis use appeared to be moderated with increasing Apfel (PONV risk) score. In terms of absolute changes in probability of PONV, daily users were predicted to have a mean increase in risk of 3.3% (95%CI 0.4–6.4%) compared to non-users, while current users were predicted to have a mean increase in risk of PONV of 1.2% (95% CI -0.7 - 3.1%).

**Fig. 3:**
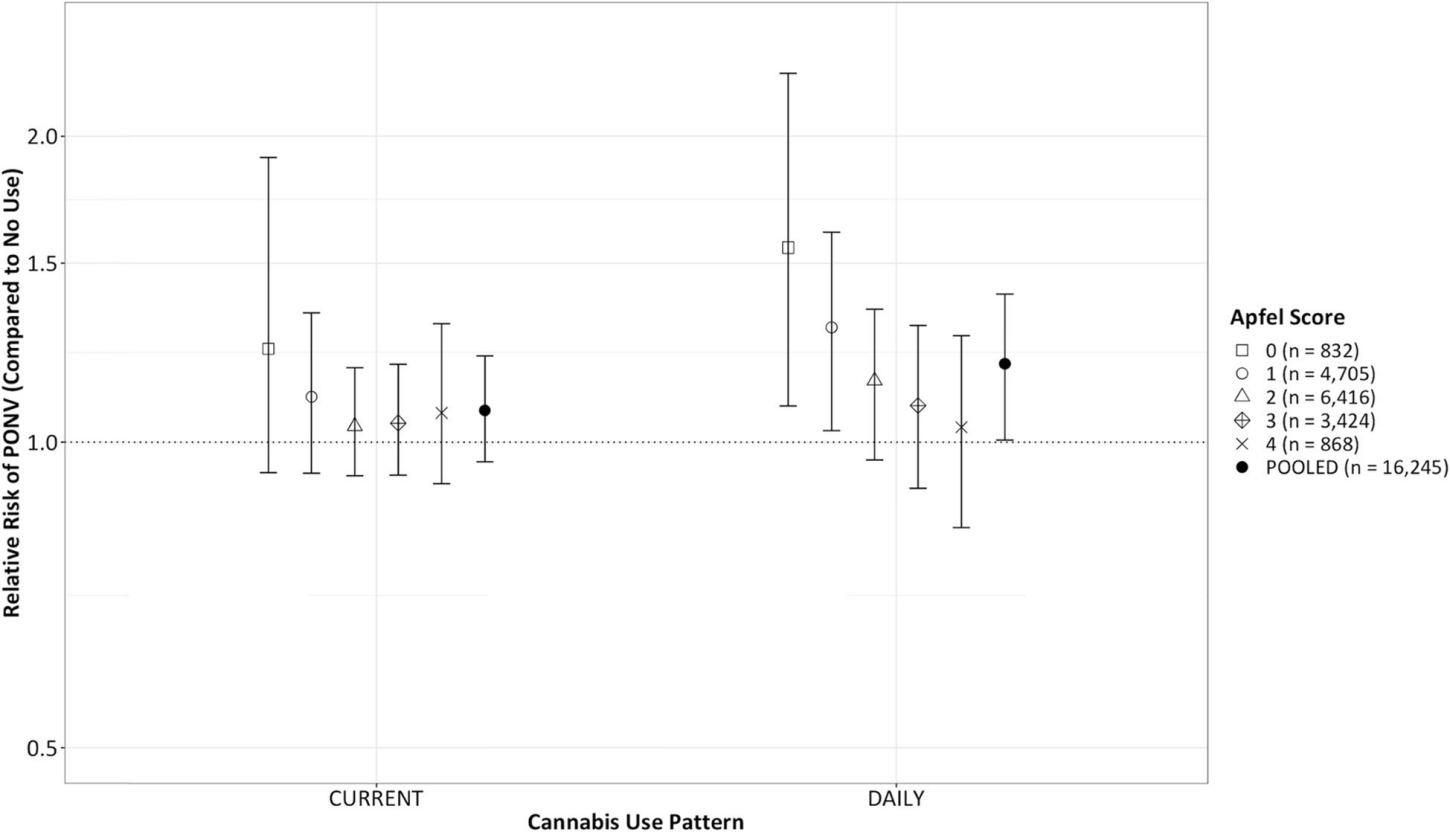
Sample average treatment effect (SATE) measured as relative risk of any postoperative nausea/vomiting modeling entire sample as non-users, current (non-daily), or daily cannabis users. Estimates stratified by Apfel score. 95% Bayesian posterior credible interval for SATE generated from 1000 MCMC estimates. Pooled estimate across all Apfel scores showed at right of each grouping

We validated our BART model’s results using two techniques: first, we compared its predictions to a Bayesian logistic regression model using propensity score adjustment (Table 2). The model’s odds ratio for daily cannabis use was 1.16 (95% CI 0.99–1.35), and the sample average treatment effect (calculated as a relative risk) was 1.13 (95% CI 0.97–1.30). We then replicated our BART modeling strategy using independently generated data at HMC (Fig. 3). We observed a nearly identical sATE at HMC, with an estimated mean relative risk of 1.19 (95% CI 1.00–1.40, posterior probability RR > 1 = 97.7%) for daily cannabis users.

**Table 2:** Odds ratios (exponentiated coefficients from Bayesian Bernoulli model) with 95% credible intervals. Probability of THC (predicted from separate BART model) modeled directly as propensity score

Model Parameter	Odds Ratio for any PONV	95%CI
Age < 50 years	1.37	1.29–1.46
ASA 1 (Reference)	1	–
ASA 2	1.02	0.92–1.13
ASA 3	0.93	0.83–1.05
**No Use of Cannabis (Referent)**	**1**	**–**
**Current Use of Cannabis (Compared to No Use)**	**1.07**	**0.96–1.18**
**Daily Use of Cannabis (Compared to No Use)**	**1.16**	**0.99–1.35**
Exposed to Nitrous Oxide	1.12	1.03–1.21
Exposed to Potent Volatile Agent	1.81	1.70–1.95
Surgical Duration (minutes, log transformed)	1.43	1.37–1.50
Female Sex	1.95	1.84–2.07
History of PONV or Motion Sickness	1.49	1.39–1.59
Non-smoker	1.67	1.41–1.92
Opioids in PACU	1.55	1.47–1.65
Per 1% Increase in Probability of THC Use (Propensity Score)	1.06	1.04–1.08
Per Prophylactic PONV Drug Given	0.87	0.84–0.89

## Discussion

This two-center retrospective cohort study identified an association between chronic cannabis use and an increased risk of postoperative nausea and vomiting. Using modern statistical techniques for estimating causal effects, we observed a mean increase in relative risk of PONV associated with daily cannabis use of 1.20, with a 95% Bayesian credible interval of 1–1.45. This estimate is supported by the fact that we calculated a nearly identical estimate in a second hospital with a different patient population and different providers. Many providers might assume that chronic cannabis use exerts some form of lasting antiemetic effect; however, our analysis indicates the potential for an increased risk of postoperative nausea among such patients. Although a dose-response effect may exist with respect to the dose of daily exposure to cannabis, this could not be tested retrospectively with the data available to us.

In our analysis, we observed that the association between cannabis use and PONV appeared to decrease with increasing Apfel score (i.e. Apfel score exerted a moderating effect in the model). One interpretation of this observation is that daily cannabis is a relatively “weak” risk factor for PONV when compared to the classical risks measured in the simplified Apfel score. While the credible intervals for relative risk begin to include 1 (no increase in risk), one advantage to Bayesian modeling is that a credible interval that includes 1 does not indicate a non-significant effect, but rather an increased probability of a non-positive (or even negative) association. We would argue that classical models for PONV may under-measure the complexity of interaction between risk factors, and advocate for statistical approaches like BART that permit a data-driven, non-parametric approach to data analysis that can reveal additional complexity.

Cannabinoids exert a well documented antiemetic effect, though it remains less clear whether they are similarly effective at preventing nausea [20, 21]. The antiemetic effects of cannabinoids are thought to be mediated by activation of CB-1 receptors in the area postrema of the nucleus tractus solitarus and the “vomiting center” of the medulla [22]. The observation that cannabis acted as an antiemetic and the intractability of nausea in patients receiving chemotherapeutic agents for cancer treatment stimulated the development of synthetic cannabinoids to specifically treat CINV. Two such synthetic cannabinoids, dronabinol and nabilone, have been shown to be effective treatments for CINV [23, 24]. In a more recent study, dronabinol was found to be as effective as ondansetron in reducing the incidence of nausea and vomiting in patients on highly emetogenic chemotherapy [25]. In that study, patients receiving dronabinol also reported decreased severity of their nausea and retching. Nabiximols (trade name Sativex), a whole plant extract of cannabis, available as an oro-mucusol spray in Canada and Europe, has been demonstrated to be superior to placebo in decreasing CINV [5, 26].

Though cannabis and synthetic cannabinoids are used to treat CINV, their use to treat PONV has not been established. In a recent randomized controlled trial, Kleine-Brueggeney and colleagues compared intravenous THC prior to emergence from general anesthesia to placebo, but the study was discontinued due to unacceptable side effects, including sedation and psychotropic phenomena [9]. In another trial comparing nabilone to placebo in patients at high risk of PONV receiving a standardized regiment of other antiemetics, the authors concluded that nabilone did not decrease the incidence of PONV [8]. Notably, nabilone also failed to improve pain scores, opioid consumption, or side effects. The use of cannabinoids for intractable PONV has also been described in a case report in which a young woman who underwent laparoscopic gastric bypass surgery experienced intractable postoperative nausea lasting weeks following surgery [10]. After multiple admissions and treatments, the patient was finally given dronabinol and experienced a significant improvement in her nausea within 1–2 days.

Paradoxically, cannabinoids can also elicit nausea, as seen in Cannabinoid Hyperemesis Syndrome (CHS), a condition associated with heavy daily use of cannabis. CHS has been theorized to be caused by either a buildup of toxic chemicals found in cannabis or the downregulation of CB1 receptors in chronic cannabis use [21]. Genetic differences in the P450 enzyme family responsible for cannabinoid metabolism may also play a role [26]. Withdrawal symptoms after cessation of chronic cannabis use include nausea, irritability, anxiety, sleep disturbances, restlessness, depressed mood, and physical discomforts such as abdominal pain, and typically begin within 24–48 h, with onset depending upon the type of cannabinoid used, the route of ingestion, and the frequency and amount of consumption. Withdrawal timing and severity may also have a genetic component. Kebir et al. recently described the presence of a polymorphism in a cannabinoid transporter which can significantly alter THC levels in the blood and body stores resulting in more severe withdrawal symptoms [27]. Interestingly, Schilienz et al. found cannabinoid withdrawal symptoms to be more severe in females, specifically nausea, though nausea was less common than other physical symptoms of withdrawal [28].

Several possibilities could explain why patients chronically using cannabis in an outpatient setting demonstrated a higher risk of developing postoperative nausea and vomiting in our study. The simplest hypothesis is that patients were demonstrating symptoms of cannabis withdrawal. While cannabis withdrawal symptoms generally take several days to appear, the exposure to emetogenic stimuli (e.g. anesthetic and analgesic drugs, peritoneal stretch) combined with reduction or abstention from cannabis use in the perioperative period might unmask withdrawal symptoms earlier than they might be expected. Another possibility is that patients using cannabis choose to do so in part because of the drug’s antinausea properties. In this conception of risk, cannabis itself is not emetogenic, but rather a marker for a patient at elevated risk of PONV who is chronically self-medicating.

Our study’s observations are strengthened by our use of a modern statistical technique for obtaining estimates for causal inference that avoids some of the classical problems associated with matching and propensity score estimates. Further, we performed both internal and external validation analyses, a process which we believe strengthens our results. As is true of any non-randomized study of an intervention, we are limited by potential associations between our predictor (cannabis use) and outcome (PONV) that are not appropriately managed by our statistical methods. We find it unlikely that a randomized study to answer this question will ever be conducted, and therefore hope that other groups with comparable datasets will explore this question and provide additional independent analyses that would provide further confirmation or spur debate as to the reliability of our findings.

## Conclusions

Patients who chronically use cannabis may be at increased risk of postoperative nausea and vomiting following general anesthesia. Further studies seeking to confirm and extend our findings could examine as to the symptoms being managed by cannabis use (if patients are using it medicinally). Furthermore, future studies would benefit from a finer grained understanding of patient’s frequency, chronicity, route, and quantity of cannabis use, as well as whether the patient has experienced symptoms during abstention from cannabis use in the past. Finally, we believe it would be inappropriate on the basis of our study alone to recommend any modification in the approach to PONV prophylaxis for the chronic cannabis user, and encourage providers to wait for further data to integrate our findings into their clinical practice.

## Supplementary information

**Additional file 1: Table S1.** Demographic and clinical data for general anesthetics at HMC. Continuous variables are summarized by mean (sd). Categorical variables are summarised by n (%). Ordinal variables are summarized by median and interquartile range

## Data Availability

The datasets generated and/or analyzed during the current study are available in the ponvthc repository, hosted at https://github.com/ponvthc/publication_dataset (10.5281/zenodo.3674310).

## Abbreviations

PONV: Postoperative nausea and vomiting
PACU: Postanesthesia care unit
BART: Bayesian Additive Regression Trees
CINV: Chemotherapy induced nausea and vomiting
UWMC: University of Washington Medical Center
HMC: Harborview Medical Center
sATE: sample average treatment effect
MCMC: Markov chain Monte Carlo
CHS: Canabinoid hyperemesis syndrome
