# Supplementary material for: Cannabis use is associated with a small increase in the risk of postoperative nausea and vomiting: a retrospective machine-learning causal analysis
Source: BMC Anesthesiol. 2020 May 18;20:115. doi: 10.1186/s12871-020-01036-4 (PMC7236204; doi:10.1186/s12871-020-01036-4)
Supplement: Supplementary file 1 — Additional file 1: Table S1. Demographic and clinical data for general anesthetics at HMC. Continuous variables are summarized by mean (sd). Categorical variables are summarised by n (%). Ordinal variables are summarized by median and interquartile range [file 12871_2020_1036_MOESM1_ESM.docx]

**Supplementary Table 1**

|  | **Cannabis Use** | | |
| --- | --- | --- | --- |
| *Variable* | **None** | **Current** | **Daily** |
| n (%) | 13392 (82.4) | 1975 (12.2) | 878 (5.4) |
| **Preoperative Data** | | | |
| Age in years [mean (sd)] | 50.43 (16.51) | 43.20 (15.13) | 43.18 (15.22) |
| ASA [n (%)] |  | | |
| 1 | 1521 (11.4) | 265 (13.4) | 97 (11.0) |
| 2 | 6332 (47.3) | 1036 (52.5) | 478 (54.4) |
| 3 | 5539 (41.4) | 674 (34.1) | 303 (34.5) |
| Outpatient [n (%)] | 5409 (40.4) | 807 (40.9) | 457 (52.1) |
| Male Sex [n (%)] | 7806 (58.3) | 1406 (71.2) | 635 (72.3) |
| Non-smoker [n (%)] | 10728 (80.1) | 1207 (61.1) | 548 (62.4) |
| Prior PONV/Motion Sickness [n (%)] | 2147 (16.0) | 285 (14.4) | 174 (19.8) |
| **Intraoperative Data** | | | |
| Procedure Duration (min) [mean (sd)] | 128 (100) | 136 (106) | 134 (104) |
| Exposed to Nitrous Oxide [n (%)] | 5304 (39.6) | 830 (42) | 384 (43.7) |
| Surgery Higher Risk for Nausea [n (%)] | 725 (5.4) | 68 (3.4) | 36 (4.1) |
| Total Number of Prophylactic Agents (median, [IQR]) | 1 [1,2] | 1 [1,2] | 1 [1,2] |
| **Postoperative Data** | | | |
| PACU Opioids [n (%)] | 7883 (58.9) | 1324 (67.0) | 587 (66.9) |
| Apfel Score (median, [IQR]) | 2 [1,3] | 2 [1,2] | 2 [1,2] |
| **Outcome** | | | |
| PONV Observed [n (%)] | 2247 (16.8) | 344 (17.4) | 168 (19.1) |

**Supplementary Table 1 Legend**Demographic and clinical data for general anesthetics at HMC. Continuous variables are summarized by mean (sd). Categorical variables are summarised by n (%). Ordinal variables are summarized by median and interquartile range
